# Economic insecurity, access to menstrual materials, and sexual coercion: a qualitative study among adolescent schoolgirls in Ibadan, Nigeria

**DOI:** 10.1186/s12978-026-02302-3

**Published:** 2026-04-17

**Authors:** Elizabeth L. Frost, Elizabeth Reed, Shira Goldenberg, Angela R. Bazzi, Olufunmilayo I. Fawole, Mobolaji M. Salawu, Omowumi O. Okedare, Georgia L. Kayser

**Affiliations:** 1 School of Public Health, San Diego State University, San Diego, CA USA; 2 Herbert Wertheim School of Public Health, University of California San Diego, San Diego, CA USA; 3 University of Ibadan, Ibadan, Nigeria

**Keywords:** Generative artificial intelligence, GenAI, Menstrual hygiene management, Period poverty, Menstrual hygiene, Menstrual health, Adolescent girls

## Abstract

**Background:**

Menstrual hygiene management (MHM) is a critical but often overlooked component of adolescent girls’ health. Limited access to financial resources for purchasing menstrual materials is a key barrier to adequate MHM. Few studies have examined how adolescent girls obtain materials, how economic insecurity may affect MHM, and whether this promotes reliance on male partners for financial means to purchase materials. Prior research has linked dependence on male partners for basic needs like food with increased risk of sexual coercion, yet little is known about whether dependence on male partners for menstrual materials carries similar risks. This study examined MHM among adolescent girls, focusing on how economic insecurity may drive girls’ reliance on male partners for menstrual materials and potential exposure to intimate partner violence (IPV).

**Methods:**

We conducted one-on-one interviews with 38 adolescent girls ages 15–19 from eight schools in low-income communities of Ibadan, Nigeria. Data was gathered on menstrual hygiene practices, access to and affordability of menstrual materials, and how relationships with male partners influence access to menstrual materials. Conversational analysis was conducted using QInsights BV to identify key themes, patterns, and insights from textual data.

**Results:**

A total of 38 adolescent girls were interviewed. Participants’ mean age was 16 years, half were currently in relationships, and 31% worked for an income alongside attending school. Emergent themes showed mothers typically provided menstrual materials or money to pay for materials. Participants also reported borrowing money from friends and relying on support from family members such as sisters, aunts, and grandmothers for menstrual materials. Some participants reported receiving money from male partners to purchase pads. Of these, several described being sexually pressured in exchange for financial support.

**Conclusions:**

Our research suggests limited access to menstrual materials may contribute to girls’ financial reliance on male partners, potentially increasing vulnerability to sexual coercion. Our study is among the first to qualitatively examine how menstrual health insecurity may foster financial dependence on male partners and, in turn, heighten vulnerability to sexual coercion. Future research and programs should address the intersection of MHM, reliance on male partners, and IPV.

**Supplementary Information:**

The online version contains supplementary material available at 10.1186/s12978-026-02302-3.

## Abstract (plain language summary)

Managing menstrual periods is an important part of girls’ health and well-being, but many girls, especially those in low-income communities, struggle to afford menstrual materials. This study looked at how teenage girls in Ibadan, Nigeria, obtain the menstrual materials they need and whether access affects their relationships or safety. We spoke with 38 girls aged 15 to 19 from eight schools in low-income areas. Most girls said their mothers usually provide pads or give money to buy them. Some girls also got help from sisters, aunts, grandmothers, or friends. A few girls said they worked to earn money for themselves. However, some girls reported relying on their boyfriends for money to buy pads, and in some cases, these boyfriends expected sex in return for the financial support. The study used qualitative data analysis in order to help identify key patterns and themes in the girls’ stories. We found that when girls cannot consistently afford menstrual materials, some turn to male partners for help, which can lead to unequal power dynamics and increase their vulnerability to sexual pressure or even sexual coercion. These findings suggest that menstrual hygiene is not just a health issue—it’s also connected to girls’ economic security and overall safety. Programs that support menstrual health should also consider how lack of access to materials may put girls at risk and should incorporate strategies to encourage girls’ economic empowerment and prevent intimate partner violence.

**Supplementary Information:**

The online version contains supplementary material available at 10.1186/s12978-026-02302-3.

## Introduction

Menstrual hygiene management (MHM) is a critical but often overlooked component of adolescent health, especially in low-resource settings where access to menstrual materials, sanitation facilities, and reliable information on menstruation may be limited. According to the WHO/UNICEF Joint Monitoring Programme, MHM is the use of clean, absorbent menstrual materials in sufficient quantity and the ability to change in a private, safe space [1]. The consequences of inadequate MHM are far-reaching, leading to adverse health outcomes including reproductive and urinary tract infections, skin irritation, and an increased risk of bacterial vaginosis, particularly among girls who are forced to prolong the use of menstrual materials due to insufficient supplies [2, 3]. Inadequate MHM also contributes to school absenteeism, as girls without access to safe, private spaces with water for changing and cleaning their menstrual materials will struggle to manage their periods effectively while at school [4–6]. This can hinder girls’ school attendance when menstruating, and thus their educational progress, and may limit future career opportunities [7, 8].

While a substantial body of research on MHM emphasize mothers, sisters and other female relatives as key providers of menstrual-related information for young girls [9–11], there remains limited evidence on how girls acquire menstrual materials, particularly regarding access and affordability. Limited access to affordable and reliable menstrual materials may be one of the more pressing barriers to effective MHM. It has been noted that in Nigeria, access to menstrual materials is a significant challenge for many, with around 37 million women and girls unable to manage their period due to financial constraints and inability to afford pads, pain medication or underwear [9, 12]. While estimates indicate that 42% to 60% of girls in Nigeria use disposable or reusable pads [9, 13], the high cost of these products can push girls to use alternative menstrual materials. For example, a study in southeastern Nigeria revealed that 40% of girls reported using rags, while 32% resorted to use toilet paper when pads were unaffordable [13].

Economic insecurity plays an important role in access to menstrual materials. Economic insecurity exacerbates girls’ inability to acquire basic necessities, particularly when girls lack both the money and the decision-making power to purchase essential items [14, 15]. This is also true for purchasing essential necessities such as menstrual materials. Previous research has established that when women and girls have access to and control over their own money to spend on needed items, their menstrual health improves. For example, one study among Ethiopian girls found that those who received pocket money from family and spent it on menstrual materials were 1.6 times more likely to practice good MHM (defined in the study as changing materials about 3–4 times per day) compared to those who did not receive any financial support from family [16]. However, more research is needed to understand if economic insecurity alone is a driver of poor MHM. Additional research is needed to explore how families allocate limited financial resources and whether menstrual materials are viewed as a spending priority. In households facing economic hardship, menstrual needs may be deprioritized, with items like pads falling lower on the list of essential household purchases.

Economic insecurity can also lead girls to rely financially on male partners — often older men who are in a social position to provide monetary support — to meet their basic needs, including access to menstrual materials [15, 17–19]. Accordingly, girls who are dependent on male partners — a term that includes boyfriends, sexual partners and romantic partners — for financial support or those who lack control over their own financial resources are at greater risk of experiencing sexual violence, including sexual coercion, particularly if the male partner expects sex in exchange for any money he provides to a girl [14, 18, 20, 21]. Dependency on a male partner for financial support can create a power imbalance within the relationship that reduces women and girl’s ability to make financial decisions, which in turn increases risk of various forms of intimate partner violence (IPV) including controlling behaviors, manipulation, isolation, and economic abuse [14]. Despite existing evidence highlighting this pathway from economic insecurity to financial dependence on a male partner and IPV [14, 20], there remains limited research exploring how this dynamic manifest in the context of MHM. Gaps in the literature point to a need for deeper investigation into the role that male partners may play in girls’ access to menstrual materials and the potential consequences of any such reliance.

To conceptualize the mechanisms linking economic insecurity, access to menstrual materials, and vulnerability to sexual coercion, this study draws on the Gender Equity in Water, Sanitation and Hygiene (WASH) framework developed by Caruso et al. [22]. The framework situates women’s and girls’ empowerment across three interconnected domains: access to resources, agency to make and act on decisions, and the multi-level enabling environment, including the social, economic, political, and physical contexts in which women and girls live. We adapted this framework to the context of menstrual health to explore how limited access to menstrual materials, combined with constrained agency and adverse social and economic conditions, can shape girls’ strategies for meeting their menstrual needs. These gender-based constraints may heighten girls’ vulnerability to rely on interpersonal relationships to access essential resources, especially when they lack independent financial means, and increase their risk for coercion or exploitation.

Drawing on this framework, the present study examines how economic insecurity, menstrual health insecurity, and financial reliance on male partners intersect to influence adolescent girls’ risk of sexual coercion. Under conditions of economic insecurity shaped by entrenched gender inequities, adolescent girls often face constrained access to education, income-generating opportunities, and control over financial resources. These constraints are reinforced by social norms that prioritize boys’ education and economic autonomy, limit girls’ mobility and decision-making power, and position males as primary economic providers. As a result, girls may lack independent means to meet basic needs, including food, transportation, school supplies, and menstrual products. Additionally, in these contexts, girls may come to rely on interpersonal relationships — particularly with male partners — to access essential resources. When such access is mediated through unequal gendered power relations, resource provision may become conditional, implicit, or explicitly tied to expectations of sexual availability, compliance, or emotional dependence. These dynamics can heighten girls’ vulnerability to coercion or exploitation, as refusal or negotiation may carry the risk of losing access to critical material support.

The aim of this study was to explore menstrual health practices and access to menstrual materials among adolescent girls in low-income communities of Ibadan, Nigeria, with particular attention to how economic insecurity shapes reliance on male partners and associated vulnerabilities to sexual coercion. The specific objectives were: (1) Describe menstrual health practices and perceived barriers to accessing menstrual materials among adolescent girls from low-income communities in Ibadan, Nigeria, as reported during in-depth qualitative interviews, (2) Explore the strategies adolescent girls use to obtain menstrual materials in contexts of economic insecurity, including reliance on male partners, as expressed in participants’ narratives, and (3) Examine how reliance on male partners for menstrual materials may shape vulnerability to sexual coercion. This study sought to advance our understanding of how adolescent girls access necessary menstrual materials and provides insights to inform future interventions aimed at improving MHM.

## Methods

This study employed a qualitative design, with quantitative survey data used descriptively to characterize the sample and complement the in-depth interviews.

### Study setting and sample

This qualitative study was conducted in Ibadan, the third most populous city in Nigeria, situated in the southwest region [23]. Ibadan is one of Nigeria’s major commercial and cultural centers, characterized by a diverse and dynamic economy. Economic activity is driven primarily by agriculture, commerce, handicrafts, manufacturing, and service industries. The city has a strong informal and small-business sector, with income generating activities that include corn milling, leatherworking, wood and metal furniture production, printing, hospitality, and a range of repair services, alongside a smaller number of modern manufacturing industries. This economic diversity shapes daily life and labor patterns in Ibadan and provides important context for understanding the lived experiences explored in this study [24]. As a rapidly urbanizing city, Ibadan consists of densely populated urban neighborhoods as well as peri-urban communities, with marked socioeconomic disparities influencing household resources, educational continuity, and health-seeking behaviors [24].

Participants in the study comprised of 38 adolescent girls recruited from eight schools in low-income communities of Ibadan. These participants were drawn from a larger randomized controlled trial (RCT) of *Girls Invest*; an economic empowerment intervention (*N* = 258) aimed at reducing IPV among adolescent girls by promoting financial literacy and healthy relationships via modules on a mobile app. More information about *Girls Invest* can be found elsewhere [25]. Adolescent girls ages 15–19 years old who had previously participated in *Girls Invest* and who had started their menstruation were eligible to participate.

### Study protocol

Purposive sampling was used to recruit participants from the *Girls Invest* parent study. We used this sampling method to collect data from a diverse range of girls based on age and experiences being in a relationship with a male partner. A research team, composed of young women from the community and trained in participant recruitment, qualitative data collection, and research ethics, led recruitment activities during the six-month follow-up time period of the *Girls Invest* intervention.

At each participating school, the research team informed eligible *Girls Invest* participants about the qualitative study and invited girls to indicate their willingness to participate. Only girls who expressed interest and met the inclusion criteria were enrolled. Recruitment from the parent *Girls Invest* study was intentionally done to leverage the rapport already established between the research team, participating girls, and school staff, including teachers and principals. Informed consent was obtained in private at the school by a research team member. Participants who were interested in joining the study signed a consent form. Those under the age of 18 brought the form home to obtain a parent’s signature. All participants gave informed consent, and minors received consent from at least one parent.

Data were collected in October 2024. Locally trained research staff conducted the one-on-one interviews in English or Yoruba at the schools in a private setting. All interviews were audio recorded and lasted about an hour. Participants were compensated with small gifts such as books, soaps or lotion equivalent to $10 USD (~ N 16,000) upon completion of the interviews. All interviews were transcribed manually by the local research team members.

The protocol was approved by the Human Research Protection Program at San Diego State University (HS-2023-0113) in the United States, and the Institutional Review Board at the Joint University of Ibadan/University College Hospital Ethics in Nigeria.

### Measures

To characterize the study sample, we used quantitative survey data collected at the 6-month follow-up of the *Girls Invest* intervention. These data were used solely to provide background information on participant characteristics and menstrual health practices, and to complement the qualitative data. Demographic measures included age, ethnicity, religion, employment status, living situation, and household food insecurity. Menstrual health-related measures included type of menstrual material used, missed school days due to period, ability to always get more menstrual materials when needed, worrying about how to get materials, access to clean water for washing materials, and amenities needed in school to help attend school while menstruating.

Primary data for this study were collected through one-on-one, in-depth qualitative interviews. For the qualitative interviews, we developed a semi-structured interview guide which included in-depth, open-ended questions exploring multiple dimensions of MHM. Topics addressed financial barriers to obtaining menstrual materials, the ability to regularly change or clean menstrual materials, and the impact of menstruation on school attendance and participation in social activities. Additional questions examined interpersonal dynamics, including participants’ relationships with male partners, financial dependence on these partners, expectations of the partners (e.g., what the partner wanted in exchange for money he provided to the participant), decision-making autonomy within romantic relationships, and prevailing social norms surrounding economic reliance on male partners. In situations where a participant reported she was not in an intimate relationship with a male partner, the interviewer probed for what the participant would do in a future relationship or what the participant has witnessed among her friends or family members who were in relationships. For detailed information, see the supplemental interview guide.

### Data analysis

We produced descriptive statistics (means, frequencies, and percentages) of socio-demographics and menstrual health practices in order to describe our sample of 38 adolescent girls. For the qualitative data, interviews were manually transcribed and translated into English by the local research team members. We then conducted the qualitative data analysis using conversational analysis through the use of QInsights BV [26], a qualitative analysis software that combines human expertise with advanced language models to assist in systematically identifying patterns and insights from unstructured data and includes generative artificial intelligence (genAI) [26]. Our use of genAI as part of the qualitative data analysis was motivated by its potential to enhance transparency, consistency, and efficiency in identifying preliminary patterns and themes across a large qualitative dataset, while preserving researcher-led interpretation and reflexive judgment.

Analysis was conducted using a conversational analysis approach in which traditional coding methods are replaced by the use of AI-supported data retrieval combined with interactive dialogue-based interpretation of the data [27]. Rather than employing a formal coding approach, conversational analysis relies on iterative, researcher-led engagement with genAI to explore themes, patterns, meanings, and relationships within the qualitative data. Conversational analysis began with our research team carefully reading all transcripts to become familiar with the interview content and ensure that participants’ voices were understood directly. We then proceeded with the following steps [28]:

1. Theme Generation: Using de-identified transcripts, we used QInsights BV to help generate preliminary themes. These initial themes served as a starting point for further exploration.
2. Question Development: Based on these initial themes and our understanding of the data, we developed targeted exploratory questions (prompts) to probe themes inductively and deductively. These prompts aimed to uncover nuances, such as unique or divergent responses, and to compare commonalities and differences among participants. Examples of prompts include: *I would like to explore [Theme X]. Please provide detailed insights on [Theme X]. What are the differences between respondents? What are similarities? What additional details or nuances did the respondent share about Theme X? Can you identify any underlying motivations behind the participant’s views on [Theme X]?* [30].
3. In-depth Exploration: Using our structured prompts, we iteratively queried the data using QInsights BV which incorporates genAI to explore each theme in greater depth.
4. Interpretation and Synthesis: We used the AI-generated content from QInsights BV as a foundation for deeper interpretation. We engaged in iterative questioning, critically examined the suggested themes and supporting quotes, and refined or discarded themes based on our expertise, familiarity with the context, and theoretical grounding.

Effective use of genAI in qualitative analysis, particularly for conversational analysis, requires an iterative and reflexive dialogue between the trained researcher and the tool [22]. At each stage, the researcher’s expertise is central to interpreting, validating, and contextualizing the data [28–31]. Throughout this process, our team cross-checked emerging themes against one another’s independent interpretations, the research literature, and our theoretical framework. The conversational analysis approach resulted in a co-constructed analysis between us as researchers and QInsights BV. This enabled deeper insights and connections that might have been overlooked with traditional coding methods [22].

Rather than replacing the researcher, this tool was used to assist in theme development and data exploration through advanced natural language processing. All analyses were guided and interpreted by the research team, with QInsights BV functioning as a support mechanism within a broader, researcher-led analytic process. In addition, QInsights BV maintains industry-leading security standards with double encryption of all data and aligns with the European General Data Protection Regulation (GDPR) requirements [32]. In addition, none of the data uploaded into QInsights BV was used for training the software’s algorithm, thus supplying another level of privacy protection.

## Results

### Sample characteristics

Our sample included 38 adolescent girls with a mean age of 15 years (range: 14–18, Std. Dev. 0.93 years). The majority identified as belonging to the Yoruba ethnic group (95%) and as Muslim (61%). Less than one-quarter of participants reported earning an income outside of their role as students. While most girls (87%) lived with both parents, 8% lived in a household with only their mother and 5% lived with other family members. Regarding household food insecurity, 32% of participants reported having to seek meals outside the home, such as at a relative’s or friend’s home, due to insufficient food available at home.

During their last menstrual period, 20% of participants reported using cloth, pieces of fabric or cotton wool, 31% used reusable sanitary pads, 41% used disposable sanitary pads, and 7% used toilet paper. In total, 51% of girls relied on reusable menstrual materials that require washing with clean water. However, when asked what resources they currently don’t have that would help them better manage their menstrual period, participants identified the following needs: access to clean water (10%), soap (14%), a private place to change materials (17%), money (10%), and pain medication (14%). Only 40% of participants said they were consistently able to obtain menstrual materials when needed and 57% reported that they worried about how to access menstrual materials. When asked what would help them attend school while menstruating, participants identified several key amenities that schools lack: a private place to change (36%), a private sanitation area with a locking door (25%), a waste bin (18%), and water in a private space for washing menstrual materials (18%) (Table 1).

**Table 1:** Socio-demographic characteristics of adolescent girls in Ibadan, Nigeria (*N* = 38)

Characteristic	*N* (%)	Missing
Mean Age in Years (range, Std. Dev.)	15 (14–18, 0.93)	0
Ethnicity		0
Yoruba	36 (95)	
Religion		0
Christianity	15 (39)	
Islam	23 (61)	
Living situation		0
Live with both parents	33 (87)	
Live with mother only	3 (8)	
Live with other family member	2 (5)	
Food insecurity		0
Ever felt hungry but had nothing to eat	20 (53)	
Ever eaten at a relative or friends house or get food somewhere else due to lack of food at home	12 (32)	
Income generation		5
Working or earning an income apart from being a student	8 (22)	
Material used during last menstrual period		9
Cloth/pieces of fabric	3 (10)	
Cotton wool	3 (10)	
Reusable sanitary pads	9 (31)	
Disposable sanitary pads	12 (41)	
Toilet paper	2 (7)	
Location used to change pads, cloth or other menstrual materials most often		10
Sanitation facilities at home	25 (89)	
Sanitation facilities at school	2 (7)	
Sanitation facilities at apprenticeship	1 (4)	
Resources needed but usually unavailable to help manage menstrual period		9
Clean water	3 (10)	
Soap	4 (14)	
Private place to change materials	5 (17)	
Money to purchase menstrual products	3 (10)	
Pain medication	4 (14)	
Menstrual material sufficiency		8
During last menstrual period did not have enough menstrual materials to change as often as needed	18 (60)	
During last menstrual period worried about how to get more menstrual materials	17 (57)	
Amenities that would help school attendance during menstruation		10
Waste bin	5 (18)	
Private place to change	10 (36)	
Water for washing materials	5 (18)	
Sanitation area with lock on the door	7 (25)	
Reliance on a male partner		0
Rely on a male partner for money to pay for menstrual materials	4 (11)	
Knows someone who relies on a male partner for menstrual materials	5 (13)	

### Themes from qualitative interviews

Our results are organized to address the three study objectives. Findings related to the first objective describe menstrual health practices and perceived barriers to accessing menstrual materials, and are complemented by demographic information obtained from the quantitative survey. To address the second objective, multiple themes emerged highlighting the role of economic insecurity in creating barriers to accessing menstrual materials, and the ways girls sought support from mothers, other family members, friends, and in some cases, male partners in order to meet their menstrual needs. The final theme addresses the third objective by highlighting participants’ reliance on male partners for menstrual materials and the ways this dependence shaped perceptions of vulnerability to sexual coercion.

### Theme 1: Menstrual health practices and perceived barriers to accessing menstrual materials or needed amenities

This section describes adolescent girls’ menstrual health practices and the barriers they face in accessing menstrual materials. Findings are organized around the following sub-themes: preferences for menstrual materials, the role of economic insecurity in limiting access, stigma and discomfort in seeking support, and challenges related to inadequate WASH facilities and limited institutional support within school settings.

#### A preference for menstrual pads

A majority of participants spoke about their preference for disposable pads over cloth due to the convenience, comfort and hygiene benefits. Participants liked the fact that disposable pads do not require washing. Participants also believed that disposable pads were more hygienic — killing germs and preventing infections. Likewise, disposable pads were believed to be more effective at preventing leakage and staining of outer clothing. Cloth was the backup option for many participants, particularly when they didn’t have money to afford disposable pads.

I don’t like cloth because one has to wash to reuse but you discard pad after every single use. Also, [cloth] shifts on the pant since it is not fixed like pads. ID4.4 (Age 17)

I like the fact that it does not give me infection because if you use cloth any fungi that enters the cloth or bacteria that enters and you’re using it with it, it can cause something bad in your body. I like to use the pad because it won’t cause anything when I use it. ID3.3 (Age 17)

#### Economic insecurity limits access to menstrual materials

Economic insecurity greatly impacted access to menstrual materials. Financial constraints often limited the ability of participants to purchase their preferred menstrual materials such as reusable and disposable pads. To mitigate economic insecurity, participants discussed setting aside money specifically for purchasing menstrual materials like pads. These savings often come from allowances provided by family members or their own earnings. Participants view saving money as crucial to avoiding the embarrassment or discomfort of relying on others for menstrual materials. Despite their efforts to save money, some participants still some participants still struggled financially to afford menstrual materials, prompting them to use alternatives like cloth. Many participants reported having savings, while those without savings rely on borrowing money from friends or family, and in several cases, participants described purchasing pads using credit from a local shop.

If there’s no money to buy pad, one can use cloth. ID4.5 (Age 17)

If there is no money for pad, I use toilet paper. ID5.2 (Age 16)

If I have been given money, I can save from it so that I don’t keep asking my family for money anytime a need arises…I do use it to purchase menstrual products. QA2.4 (Age 15)

I save the money. When I need to buy pad, I take from it. ID3.1 (Age 18)

If there is no money on me and if my mum is around, I can borrow money from her to buy pads or if the woman selling it [at the local shop] is also around, I can get the pad from her on credit and when my mum is back in the evening, I will collect money from her to pay my debt. ID7.2 (Age 17)

Maybe I don’t have money to buy pad, I usually go to her [my mum] to borrow money and I pay her back. Also, I go to collect pad on credit from the shop opposite our house to pay later. ID6.4 (Age18)

#### Stigma and discomfort hinder girls from asking for help with MHM

Participants highlighted pervasive social stigma associated with discussing menstruation and other natural bodily functions. Many participants expressed feeling uneasy seeking information about menstruation or asking for help acquiring menstrual materials like cloth or pads. This discomfort prevents girls from getting the support they need from family, friends, or community members. For example, one participant shared that she felt too shy to tell anyone except her sister about using family-provided money to buy menstrual supplies. This reflects a widespread feeling of unease when it comes to discussing menstrual needs openly with anyone beyond close family members. However, one participant recognized that while it feels awkward, if she doesn’t ask for help with her menstruation, then she will not receive menstrual advice, money to pay for pads, or clean cloth.

No, I don’t tell the person who gives me money that I used it to buy pads, except from my sister. I am usually shy to other people. ID6.5 (Age 17)

To get the menstrual products I usually need, I buy. Nobody gives me [money or menstrual products] because I am usually shy to ask someone for something, I don’t go to anybody. I didn’t talk to anyone. I have never gone to anybody to ask. I keep things to myself…Who gives me money? Myself. Nobody buys me materials or gives me money to buy materials. I look for money to buy pads. ID3.2 (Age 17)

I do not feel comfortable but if I do not ask, nobody will help me. And this is because I do not like to tell people around me what am going through. ID6.2 (Age 17)

#### Insufficient access to WASH amenities in school

Participants frequently discussed menstrual health challenges within the school setting, as it is where they spend the majority of their day. The majority of participants stated that schools lack the necessary amenities to support girls who are menstruating, including insufficient water for washing soiled cloth or stained school uniforms, and the absence of private, secure spaces to change menstrual materials. There was also mention of restricted access to WASH services because bathrooms are often locked. Inadequate WASH access for MHM at school forced one participant to seek an alternative location, such as a friend’s home nearby, to change or clean herself. In contrast to the school setting, participants preferred to stay at home where they had continuous access to essential hygiene resources like soap and clean water, which made it easier to manage their period, especially for those using cloth to manage their menstruation.

When at school, some ladies get stained and there won’t be water facility in school for them to wash the stained uniform. ID3.1 (Age 18)

Staying at home where I can get soap and clean water to take care of myself [would help me better manage my menstruation]. ID4.2 (Age 17)

If I start my period unexpectantly, I will use water to clean my clothes. If I don’t have money, I will borrow money to buy pad and change at my friend’s house since they lock the school toilets. ID1.6 (Age 15)

#### Limited institutional support for managing menstruation within school settings

In this study, school-based support refers to institutional or staff-provided assistance available to girls when menstruation occurs during the school day, including access to menstrual materials, emergency support, and practical accommodations. Only three participants reported receiving MHM support while at school. One participant mentioned school visitors who handed out reusable pads for all the girls to use, while another participant stated that a teacher once lent money for her to purchase a pad. Receiving support from a teacher appeared to be rare. One participant shared that she received education on menstruation but never got support in obtaining pads or cloth.

Some people came around to our school last month and gave us a material that is like a pad but washable. I also have a “diva pad” [a brand of sanitary pad] then, so I used the two during my last period. ID6.4 (Age 18)

If I start my period while at school unexpectedly, I can go and meet our class teacher and tell her that I saw my period now and maybe I don’t have money on me that she should give me money that I’ll bring it for her the next day. ID6.3 (Age 17)

I buy [menstrual materials] by myself from the money I saved. My daddy buys for us sometimes. Our female teachers provide us with menstrual counselling and not pads. Nobody has ever gifted me a pad. ID3.6 (Age 15)

### Theme 2: Strategies for obtaining needed menstrual materials

This section explores the strategies adolescent girls use to obtain menstrual materials in the context of economic insecurity. Findings are organized around the following sub-themes: reliance on mothers for menstrual materials and guidance, support from other family members, and assistance from friends, particularly in situations where menstruation occurs unexpectedly or financial resources are limited. 

#### Mothers are the primary providers of menstrual materials or money for materials, and advice or guidance for MHM

Participants shared that mothers were often the primary source of menstrual materials, and guidance or emotional support during menstruation. In most cases, mothers of participants gave cloth or pads directly or provided their daughters money to purchase menstrual materials. Mothers were also reported to offer advice, create a safe space for open conversations about menstruation, and educate their daughters on using pads and managing their menstrual period effectively.

My mum knows about [which menstrual materials to use], so she always guides me right, I feel comfortable because she’s my mother. My mother always buys [menstrual materials] and keeps it at home for our use. ID8.1 (Age 15)

My mom taught me. She gave me pads and hand sanitizers to be using. Then she also gave me perfume to be using. She also enlightened me on what not to eat during menstruation. That I shouldn’t eat groundnut; it makes people smell during menstruation. That I shouldn’t take sweets also it makes some people’s flow to be heavy. ID4.1 (Age 18)

I collect money from mum and if she is not around, I can remove money from her goods sold and when she is back, I will tell her I used from her money to buy the material I need. ID8.2 (Age 18)

It is only my mum that do provide me with money to buy what I need. Sometimes, if I don’t have money, my mum can tell me to use cloth. ID1.2 (Age 16)

[My mother] supports me. She does not want me to be dependent on another person. She supports me. In fact, if [money] remains on her card, she will give me. She does not want me to be dependent on another person or I should now go and meet one boyfriend [and say] please for God’s sake give me one. ID3.3 (Age 17)

However, not all participants reported being able to rely on their mother for menstrual health support. Two participants stated that they didn’t feel comfortable asking their mother for help; one participant mentioned she didn’t want to be a burden, and the other participant perceived her mother to be unapproachable and emotionally unavailable to address her questions or concerns about menstruation. Participants’ concerns appeared to stem from stigma in asking their mothers for financial help or other forms of support.

No, I don’t feel comfortable asking [my mother] for support. I do look at my mum’s financial capacity…I don’t like to disturb her…I ask her only if I need money. Though, I do look at [my mother’s financial] situation before I can request [money] for help from her. ID6.1 (Age 16)

I don’t ask for any form of support from my mum regarding menstrual period. My mum is a disciplined person, so I cannot even ask her anything about menstruation or tell her I’m having stomach pain…No. I don’t even have the courage to ask my mum for anything, I get scared asking her for anything. ID7.2 (Age 17)

#### Other family members provide money, advice or guidance for MHM

Some participants highlighted the supportive roles of various family members, other than their mother, including fathers, sisters, brothers, and extended relatives, in helping meet their menstrual needs. The majority of participants reported that support with their menstruation was often provided by multiple family members rather than a single individual, with family members making financial contributions whenever possible. Some participants mentioned that their father gave them money to purchase pads, while others reported receiving financial assistance from a brother. About a third of participants shared that a sister often provided money to buy pads or supplied them directly, along with providing guidance and shared experiences to help younger siblings navigate menstruation. Although less common, extended family members such as aunts or grandmothers were also noted as sources of financial support or advice. The degree of involvement and assistance from family members varied widely depending on individual family dynamics.

The money my daddy gives to me…I do not usually spend everything. When I save the remaining money, I use some to buy a pad. ID2.1 (Age 17)

My sister doesn’t buy pads for me but gives me money when I ask her. ID2.2 (Age 16)

I ask my grandma for support and advice [about menstruation]. She gives me money for pad even before I see my flow. My family is very supportive. Sometimes I even forget that I don’t have pads. It’s my grandma that ask to remind me if I still have pads…She is a woman like me and also a parent. [The participant lives with the grandmother who is the main caretaker and provider]. ID4.1 (Age 18)

My brother gives me money when he returns home. My boyfriend also gives me money and I can buy pad with the money. My mummy and my sister too. I only tell them to give me money but I don’t tell them that I want to use it to buy pad. If my sister is at home, I can tell her to give me money for pad. Also, I can tell the shop where I usually buy pad on credit. ID6.4 (Age 18)

#### Friends play a supportive role with menstruation by providing pads when participants were not expecting to start their period while at school

Participants mentioned that a lack of menstrual materials at school left them to rely on their social network to access support and needed materials. When at school, friends provided emotional encouragement, shared resources (e.g., pads, cloth, money), and offered practical assistance when needed. Friends lent money to buy pads or shared their own supply of menstrual materials. This support was crucial, especially for participants whose family’s financial support was limited, for girls who had irregular periods, or for those whose menstruation started unexpectedly. However, this support from friends appeared to be informal and based on immediate necessity rather than a regular source of assistance.

If I start my period while I’m at school and didn’t expect it, I will go to the toilet to clean myself with water, give my friend money to help me buy pads. We are not allowed to go home until closing hour. ID3.5 (Age 14)

I borrow [menstrual materials] from my school friends. It has happened to me twice this year, but I’ve never bought pads on credit before. ID7.1 (Age 17)

If I start my period when at school and didn’t expect it, I will borrow money from my friend in school to buy pad and I will clean myself up. ID7.4 (Age 16)

I don’t receive help from people except from the only friend I have in school. Sometimes, she gives me money to buy pads, and she could drop pad in my bag without my notice…for example if I don’t come with money to school, she can give me money. If she does not come to school with money too, I help her. If her period shows unexpectedly and she does not have pad, I give her and vice-versa. We help each other. ID7.2 (Age 17)

### Theme 3: Reliance on a male partner or boyfriend

This section examines adolescent girls’ reliance on male partners for financial support to obtain menstrual materials and the perceived risks associated with such dependence. Findings are organized around two sub-themes: indirect financial support from male partners used to purchase menstrual materials, and perceived vulnerability to sexual coercion linked to reliance on male partners for financial support.

#### Receiving money from a boyfriend or male partner and using it to purchase pads

Several participants reported that boyfriends contributed financially, providing financial resources that were used for purchasing menstrual materials, although this support was often indirect and not specifically earmarked for such purposes. Participants shared that they received money from a boyfriend which they then used to pay for pads. Participants who took money from a boyfriend did not tell him that the money would be used for menstrual materials.

My brother, father and my boyfriend give me money. Sometimes, I can use it to buy what I need. If the money is available at the time I need to purchase menstrual material, I can use it…I will not feel comfortable to tell the person or my boyfriend that I used the money to purchase menstrual products… It is inappropriate for the boyfriend to give the girlfriend money to buy pad when they are not yet married. ID1.3 (Age 16)

My boyfriend alone gives me money for pad…When I buy, I will not tell him I have bought pad or else he will not give me money for the pad again…I ask for money even though I don’t tell him the money is for pad. ID3.1 (Age 18)

Apart from mummy and my sister, my daddy gives me money to take care of myself and I sometimes use it to buy pad or anything I want. I may use the money my boyfriend gives me to buy pad, not that he has specifically given me money to buy pads before. ID6.5 (Age 17)

I don’t ask for money from him [the male partner] to buy pads but he sometimes gives me money to buy whenever I visit him and get stained at his place. ID7.5 (Age 16)

She [my friend] told me when she doesn’t have money to buy pads she would ask for money from her boyfriend and I told her what about her mum, she said her mum doesn’t have [money] that is why she asks [for pads] from her boyfriend. ID5.2 (Age 16)

#### Reliance on a male partner and vulnerability to sexual coercion

Among participants who reported receiving financial support from their boyfriends, some mentioned fear of being pressured into unwanted sexual activities. In several instances, participants reported experiencing IPV, specifically sexual coercion where the boyfriend pressured them for sex in exchange for money he provided. The money provided was sometimes used by the participants to purchase menstrual materials but also used to purchase other items such as food, clothing, or other small gifts.

My boyfriend gives me money… [in return] he pressures me to have sex with him but I usually tell him it’s not yet time…he pressures me [to have sex] every Sunday when I visit him. ID7.5 (Age 16)

My boyfriend also gives me money and I can buy pad with the money…he pressures me, saying that he gives me money for clothes and shoes and what if he doesn’t get married to me, what would be his gain? ID6.4 (Age 18)

…anyone that collects money from a guy, the guy will ask for something bad [sex in return for money] from her. ID6.3 (Age 17)

[Talking about a friend who receives money from a boyfriend to buy pads] My thought is that maybe as he buys it for her they might have had something together [sex] that’s why he buys [pads] for her. ID7.3 (Age 16).

Participants’ accounts suggest that the need to obtain menstrual materials did not occur in isolation, but rather intersected with broader patterns of economic dependence on male partners. Participants reported that accepting money from a boyfriend, whether explicitly for menstrual materials or for general expenses, created relational expectations that constrained their ability to refuse sexual advances. Several participants described how financial support, initially perceived as assistance, later became a source of leverage within the relationship. In this context, menstrual material insecurity functioned as a catalyst for dependency, situating participants in relationships where refusal of sex risked withdrawal of financial support needed to meet basic menstrual needs.

## Discussion

This study sought to examine menstrual health practices and barriers to accessing menstrual materials among adolescent schoolgirls in low-income communities of Ibadan, Nigeria. It draws particular attention to the role of economic insecurity and dependence on male partners, and the potential implications for increased susceptibility to sexual coercion among girls. We found that to meet their menstrual needs, girls rely on a network of support that includes family and friends, and in some cases, male partners — both romantic and sexual partners. The findings also reveal substantial gaps in adolescent girls’ ability to manage their menstruation effectively within school environments, including the absence of private spaces for changing, limited access to menstrual materials, locked bathrooms, and inadequate support while at school. This study underscores the potential harms of financial dependence on male partners, including the use of such financial support to obtain menstrual materials which at times included girls being coerced or pressured into sexual activity in exchange for money to pay for pads.

Girls in our study reported obtaining menstrual materials by navigating through a support system comprising of family, friends, and boyfriends who provide money to purchase menstrual materials or directly supply the necessary items. Among those identified in their support system, mothers played a significant role. This is consistent with previous research that highlights the mother’s importance in addressing the daughters’ menstrual needs [9, 11, 33]. However, previous research notes that mothers can, at times, have limited or inaccurate MHM information [9, 34]. Given their central role, future interventions should consider equipping mothers with comprehensive knowledge about menstruation, teaching them communication strategies to discuss menstrual health with their daughters, and foster an open dialogue that reduces stigma and misconceptions.

Our findings also suggest that fathers and other male family members play a meaningful role in girls’ social support systems, either by providing — or potentially withholding — financial resources to pay for menstrual materials. Although we did not observe explicit denial of financial support by fathers, many girls reported concealing how their pocket money was used, particularly when it was spent on menstrual needs. This concealment may be driven by stigma surrounding open discussion of menstruation and may influence whether fathers and other male family members recognize or prioritize menstrual materials as essential household expenditures. This pattern reflects broader gendered norms around financial decision-making, where men’s control over household resources may shape girls’ access to essential menstrual materials [35, 36]. As such, these findings suggest that future menstrual health interventions might benefit from actively involving male heads of household, given their role in determining household expenditures.

We also found that girls leave school premises, or stay at home, to manage their period, underscoring, a crucial but often overlooked aspect of MHM — the intersection of infrastructure availability and actual accessibility. While many MHM interventions focus on the presence of sanitation facilities, fewer studies interrogate the real-world usability of these spaces for MHM. Our findings align with previous research indicating that poor school water, sanitation, and hygiene infrastructure to manage MHM is a significant problem. For instance, research from sub-Saharan Africa and South Asia has documented instances where school restrooms are either locked, poorly maintained, or lack essential resources such as water, soap, and disposal bins, limiting girls’ ability to manage their period [10]. Similarly, among a study sample of adolescent girls from Anambra State in Southeast Nigeria, 51% reported being unable to change at school due to a lack of functional toilets or changing rooms, and those who did manage to change, often had to do so in unsafe and unhygienic locations, such as bushes or unused spaces [13]. The inability to access school restrooms due to institutional policies or structural barriers may contribute to school absenteeism, discomfort, and potential health risks such as prolonged pad use, increasing the likelihood of infections [6, 37]. Future research should move beyond measuring only infrastructure presence and instead assess when, how, and under what circumstances these facilities are available and used by girls who are menstruating. Additionally, school-based interventions should work toward policy solutions that ensure restrooms remain accessible throughout the school day. Understanding the nuanced barriers to MHM accessibility is critical for designing interventions that effectively address the needs of girls and promote gender-equitable education environments.

In our study, male partners emerged as a noteworthy source of support for girls’ MHM, with 11% of participants reporting that they relied on a male partner for money to purchase menstrual pads, and 13% indicating they knew someone who had done the same. Despite its relevance, adolescent girls’ financial dependence on male partners for menstrual materials remains largely understudied. This study contributes to a limited but growing body of research that examines how adolescent girls obtain menstrual materials through financial support from male partners, and the power dynamics that underlie these exchanges.

Our findings are consistent with prior survey data from a broader sample of 258 adolescent schoolgirls in Ibadan, Nigeria, where 7% reported dependence on a male partner for menstrual materials. In addition, our findings also align with studies conducted in rural Kenya where among girls aged 12–29 it is estimated that 1.3% of participants exchanged sex for menstrual materials [38]. In another example, a study of Kenyan girls aged 14–16 described participants receiving money from boyfriends to purchase pads without disclosing the intended purpose of the funds [39]. Taken all together, these findings suggest that reliance on male partners for menstrual materials is not merely an individual coping strategy, but a patterned response to economic insecurity and limited access to financial resources within a gendered social context. It also suggests that menstrual material insecurity operates as a structurally embedded form of economic vulnerability that reshapes adolescent girls’ relationships and encourages dependency on male partners. Because menstrual needs are recurrent, time-sensitive, and often concealed due to stigma, girls with limited access to independent financial resources face constrained options for meeting these needs. In this context, reliance on male partners emerges not simply as a financial strategy, but as a response to intersecting limitations in access, agency, and institutional support.

Within the context of economic insecurity, sexual coercion emerged as a key risk associated with reliance on male partners for menstrual materials. These findings parallel those from other studies that have identified reliance on a male partner as a risk factor for IPV, including sexual coercion [14–17, 20, 40]. In our study, some participants described firsthand experiences of sexual coercion in exchange for financial support, while others spoke about such experiences as occurring to peers or “others” in their social networks. Given the sensitive nature of sexual coercion and the stigma surrounding its disclosure, indirect or second-hand accounts are a well-recognized feature of qualitative research on gender-based violence, particularly among adolescents. Such narratives can reflect a protective strategy that allows participants to discuss taboo or distressing experiences without direct self-disclosure, while still conveying shared social realities and perceived risks. This pattern of reporting has also been documented in prior qualitative studies in Kenya [39, 41], where adolescent girls more readily described coercive or transactional sexual experiences as happening to peers rather than to themselves even as these accounts revealed pervasive gendered power imbalances and economic vulnerability.

These narratives of sexual coercion highlight how the recurring and stigmatized nature of menstrual needs can intensify dependency on male partners, reducing girls’ ability to negotiate consent and increasing vulnerability to coercion. Sexual coercion thus emerges not as an isolated experience, but as a consequence of material insecurity operating within unequal gendered power relations. While our study offers important insights, further research is needed to explore whether this pattern of reliance on male partners for menstrual support and sexual coercion is present in other low-income settings where access to affordable materials remains limited.

Drawing on our adapted Gender Equity in WASH framework, the pathway explored in our study can best be understood as a convergence of restricted access to essential menstrual materials and constrained agency within an environment that oftentimes discriminates against girls and young women. When access to menstrual materials is mediated through male partners, financial support may become entangled with relational expectations, shifting power dynamics in ways that limit girls’ ability to refuse unwanted sexual advances. Sexual coercion, in this sense, is not an isolated outcome, but a risk situated within economic dependency which is shaped by gender norms and material insecurity.

To examine these complex and intersecting pathways linking economic insecurity, gender norms, and relationships, we adopted a conversational, AI-assisted approach that first identified themes followed by querying of the data for a more in-depth exploration. This methodological choice represents a novel contribution of the study. Although the use of genAI in qualitative research is rapidly emerging, most current applications — including recently introduced AI features in widely used software such as NVivo and MAXQDA [42, 43] — largely remain embedded within conventional coding paradigms, supporting tasks such as summarization, code generation, or chat-based querying of previously coded data. By contrast, our approach leveraged AI as an analytic partner to identify themes and used prompts to explore differences, similarities and nuances among respondents, enabling a more holistic examination of relational and structural dynamics. Emerging evidence suggests that genAI-supported qualitative analysis can achieve levels of accuracy and analytic rigor comparable to traditional manual coding when used interactively and reflexively [28, 44]. By leveraging this dialogic process, our approach retained interpretive depth while capitalizing on the efficiency and pattern-recognition capabilities of genAI, offering a methodological contribution to evolving qualitative analytic practices [45].

However, there are certain limitations and caveats of genAI in qualitative research that warrant attention. The use of a genAI tool is not entirely without limitation due to inherent bias in the training data that was used to develop the algorithm [45–47]. The genAI tool also lacks the ability to fully comprehend cultural and social nuances and has been known to hallucinate or fabricate data. These limitations have been minimized by the research team being well-versed in menstrual health, WaSH access, gender inequality, and IPV research literature [30], employing human oversight to validate AI-generated themes and to make the final interpretations [45], interacting with AI by using high-quality prompts to make queries [45, 48, 49], and understanding personal biases and practicing reflexivity when interpreting AI results [50]. As qualitative research increasingly addresses complex and large-scale social phenomena, with increasing sample sizes, the incorporation of genAI offers a valuable complement and tool to conventional methodologies, enhancing both rigor and scalability in data interpretation, and reduces the time necessary to run thematic analysis.

While this study is among the first to qualitatively examine how menstrual material insecurity may foster financial dependence on male partners and, in turn, heighten vulnerability to sexual coercion, it is important to acknowledge other limitations. The findings are not generalizable to all adolescent girls in Nigeria or other low-resource settings. The sample size, though sufficient for thematic analysis, may not capture the full diversity of experiences across different socioeconomic, cultural, and geographic contexts. In addition, social desirability bias may have influenced participants’ responses, particularly when discussing sensitive topics such as financial reliance on male partners for menstrual materials. Some girls may have underreported or omitted experiences due to shame or fear of judgment. Another important factor to consider is potential bias coming from the participants having previously participated in the *Girls Invest* intervention which took place before our one-on-one interviews were conducted. Through the *Girls Invest* intervention, our participants would have completed training modules teaching them about financial literacy and the importance of establishing savings, and healthy relationships. Therefore, the responses we received as part of this qualitative study should be framed in context of girls previously having undergone participation in a girls’ empowerment intervention. However, the value of our findings is not diminished by the girls’ participation in the *Girls Invest* intervention and, therefore does provide us with important information on challenges accessing menstrual materials for schoolgirls in Nigeria.

## Conclusion

This study highlights the multifaceted challenges adolescent girls in Ibadan, Nigeria face in managing their menstruation, emphasizing the complex interplay of economic, social, and structural barriers. Our findings reinforce the importance of social support systems — primarily mothers but also other family members, friends, and in some cases, male partners — in ensuring access to menstrual materials. However, reliance on male partners for financial assistance to purchase menstrual materials introduces significant vulnerabilities, including increased risks of sexual coercion and IPV, topics that remain underexplored in the MHM literature. Additionally, our study underscores the limitations of school-based WASH facilities, where the mere presence of restrooms does not guarantee accessibility, leading some girls to leave school premises or miss school to better manage their periods when restrooms are locked or menstrual materials are not available. These study findings call for targeted interventions that address MHM within the broader context of economic insecurities, gender norms, and school infrastructure accessibility. Future research should investigate the extent of financial dependence on male partners for menstrual materials and the implications for girls’ health and well-being. Moreover, interventions should be aimed at improving access to menstrual materials at school, improving access to continuously available WASH services for MHM, and reducing the stigma surrounding menstruation, particularly shame coming from within a girls’ social support system. Addressing these menstrual health challenges holistically is crucial for fostering an environment where girls can manage their periods safely, free from violence, and with dignity, without compromising their education or personal well-being.

## Supplementary Information

Supplementary Material 1.

## Data Availability

The data used and/or analyzed during the current study are available from the corresponding author on reasonable request.

## Abbreviations

GEN AI: Generative artificial intelligence
IPV: Intimate partner violence
MHM: Menstrual hygiene management
UNICEF: United Nations Children’s Fund
WASH: Water, sanitation and hygiene
WHO: World Health Organization
