# Supplementary material for: Economic insecurity, access to menstrual materials, and sexual coercion: a qualitative study among adolescent schoolgirls in Ibadan, Nigeria
Source: Reprod Health. 2026 Apr 17;23:119. doi: 10.1186/s12978-026-02302-3 (PMC13267590; doi:10.1186/s12978-026-02302-3)
Supplement: Supplementary file 1 — Supplementary Material 1. [file 12978_2026_2302_MOESM1_ESM.pdf]

INTERVIEW GUIDE

Menstrual hygiene practices

1. Before you had your first menstrual period, what did you know about menstruation? Can you describe your experience learning what materials to use and getting those materials?

2. During your last menstrual period, what menstrual materials did you use: Cloth/reusable pads, disposable pads, tampons, menstrual cup, toilet paper, underwear alone, mattress pads, other materials (please specify)?
    a. If you used cloth, where do you get the cloth from *(Prompt: cloth from shirts/dresses, scraps of old cloth, old blankets, new cloth from the market?)*

3. Of all the different menstrual material options, which one would you prefer if you could choose anything you wanted? During your last menstrual period, were you able to use your preferred type of menstrual materials throughout your period? Why or why not?

4. What do you like or dislike about the menstrual materials you use?

5. During your last menstrual period, did you have enough menstrual materials to change them as often as you wanted to? If not, what did you do?

6. What do you think are some of the things that make it challenging for young women to manage their period?

7. During your last menstrual period, did you have to miss out on any social activities or were restricted in doing any activities because of your menstrual period? (*Prompt: going to school, going to your apprenticeship, cooking food for others, eating with others, bathing in regular place?*) Can you tell me more about that?

8. What happens if you start your period while you are at school and you didn't expect it? What do you do?

9. Do you worry about how you will meet your menstrual needs in the future when you are done with school and have a job? Can you tell me more about that?

Access to menstrual products

10. How do you get the menstrual materials you usually use?

11. When you need pads, cloth or other materials, where do you usually get them from?

12. Do you feel comfortable asking for support such as advice, resources, emotional support for your menstrual period if you need it? Why or why not? Who do you go to when you want to talk about your period? (*Prompt: Mother, friend, someone else?*)

13. How do you know where to get materials from?

14. Who is the main person who provides you with materials or money to buy materials? *(Probe: Parents, family member, boyfriend/male partner, friends, teachers, guardian?)*
    a. Does that person give you money to purchase the pads/cloth/other materials?
    b. Does that person directly give you the pads/cloth/other materials?
    c. Does that person ever ask for anything in exchange?

15. Do you receive help from friends, parents, other family members or anyone else to get materials you need? Who helps you? How do they help you?

16. How supportive is your family to make sure you have items you need during your menstrual period?

17. Has there ever been a time when you weren't able to get pads or other materials? Why did this happen? What did you do when this happened? How often does this happen?

18. Do shops or kiosks have pads that you can purchase? Do you go to the shop to purchase pads? Why or why not?

Paying for products

19. How do you usually pay for menstrual products?

20. Does anyone give you money? *(Probe: Family, friends, boyfriend, another person?)* What do you use this money for? Do you use this money to purchase menstrual materials? Do you tell that person that you are using the money to purchase menstrual materials? Why or why not?

21. Do you currently have money saved? Why or why not? If yes, what are you saving for? Do you use this money to purchase menstrual materials? What menstrual materials do you usually buy with the money you have saved?

22. Do you owe any people money? If so, how does this impact your ability to get menstrual materials?

23. During the last school term, did you need to buy menstrual hygiene products, but not have enough money? Can you tell me more about this? What did you do to manage your period?

24. What would help you better manage your menstruation? *(Probe: More information about menstruation, support from family or friends, access to materials, access to pain killers, a private area to change your menstrual products, clean water, etc.)*.

25. What are your suggestions to make it easier for young women to obtain materials for their period?

26. What are your suggestions to make it easier for young women to engage in activities such as attending school, visiting with friends, doing an apprenticeship when they are menstruating?

Relationships and menstrual periods

For participants who are in a relationship or have had a relationship

27. Does your boyfriend or male partner give you money? What do you spend it on? Does your boyfriend or male partner ask for anything in return? Does your boyfriend or male partner pressure you to do things you don't want to do? What do you do when this happens?

28. Who decides how you spend your money and manage your financial affairs? How are these decisions made between you and your boyfriend?

29. Who in the relationship should decide on spending money to purchase menstrual products? Why?

30. Have you ever asked a boyfriend or male partner for pads or money to buy menstrual materials? What was that experience like? What did you give him in return?

31. Do you know of anyone who has ever received money from a boyfriend or male partner to buy menstrual materials? Do you know anyone who received pads from a boyfriend or male partner? What do you think about this? Does the male partner or boyfriend ask for anything in return or pressure that person into doing things they don't want to do?

For participants who have never been in a relationship

32. Think about future relationships (not marital/marriage). Who in the relationship should decide how money is spent? Who should be the one to manage financial affairs? Who should decide on spending money to purchase menstrual products? Why? How are decisions about spending money different if you are married?

33. Do you know of anyone who has ever received money from a boyfriend or male partner to buy menstrual materials? Do you know anyone who received pads from a boyfriend or male partner? Can you tell me more about this? What did she give him in return? Does the male partner or boyfriend ask for anything in return or pressure that person into doing things they don't want to do?
